# Green chromatographic approach to determine methocarbamol, aspirin and their related impurities in their combined pharmaceutical formulation and human plasma: with computational evaluation

**DOI:** 10.1186/s13065-025-01500-7

**Published:** 2025-05-21

**Authors:** Yasmine F. Bassuoni, Asmaa M. AboulMagd, Maha M. Ibrahim

**Affiliations:** 1 Pharmaceutical Analytical Chemistry Department, Faculty of Pharmacy, Modern University for Technology and Information (MTI), 12582 Al Hadaba Al Wosta, Cairo, Egypt; 2 Pharmaceutical Chemistry Department, Faculty of Pharmacy, Nahda University in Beni-Suef (NUB), Beni-Suef, 62513 Egypt

**Keywords:** Methocarbamol, Aspirin, Guaifenesin, Salicylic acid, HPLC, Green analytical approach, Whiteness assessment

## Abstract

Nowadays, one of the main objectives is to protect the environment and reduce the adverse impacts of chemicals on it. One of the most difficult issues for researchers is determining the efficiency and quality of the suggested approach in terms of environmental and human safety. The present research presents advanced, accurate, green high-performance liquid chromatographic method (HPLC) to estimate a combination of methocarbamol (MET) and aspirin (ASP) as well as their related impurities, guaifenesin (GUF) and salicylic acid (SA), respectively, with low retention time and ideal separation. Chromatographic separation was performed on X-Bridge C_8_ reversed phase (RP) column (4.6 × 250 mm, 5 μm particle size) utilizing methanol: water: triethylamine (70:30, 0.1% by volume) at pH 3.00 by o-phosphoric acid as a mobile phase with 2.00 mL min^−1^ flow rate at 254.00 nm. The proposed method succeeded to achieve linearity over a concentration ranges of 0.30–50.00 μg mL^−1^, 1.00–300.00 μg mL^−1^, 10.00–500.00 μg mL^−1^ and 0.10–50.00 μg mL^−1^ for GUF, MET, ASP and SA, respectively. All the developed results were validated in compliance with the International Council of Harmonization (ICH) instructions. The suggested method was successfully applied to assess MET and ASP in their pharmaceutical formulation and in spiked human plasma. The molecular docking approach was employed to evaluate how the two drugs and their related impurities interacted with the stationary phase and to predict the suggested sequence of elution. The greenness of the developed analytical method was evaluated using whiteness assessment and two green approaches; analytical eco-scale and AGREE. The proposed method proved not only to be green, time-saving and more sensitive for the routine analysis of MET and ASP in QC laboratories, but also it can effectively handle the presence of impurities such as GUF and SA.

**Graphical Abstract:**

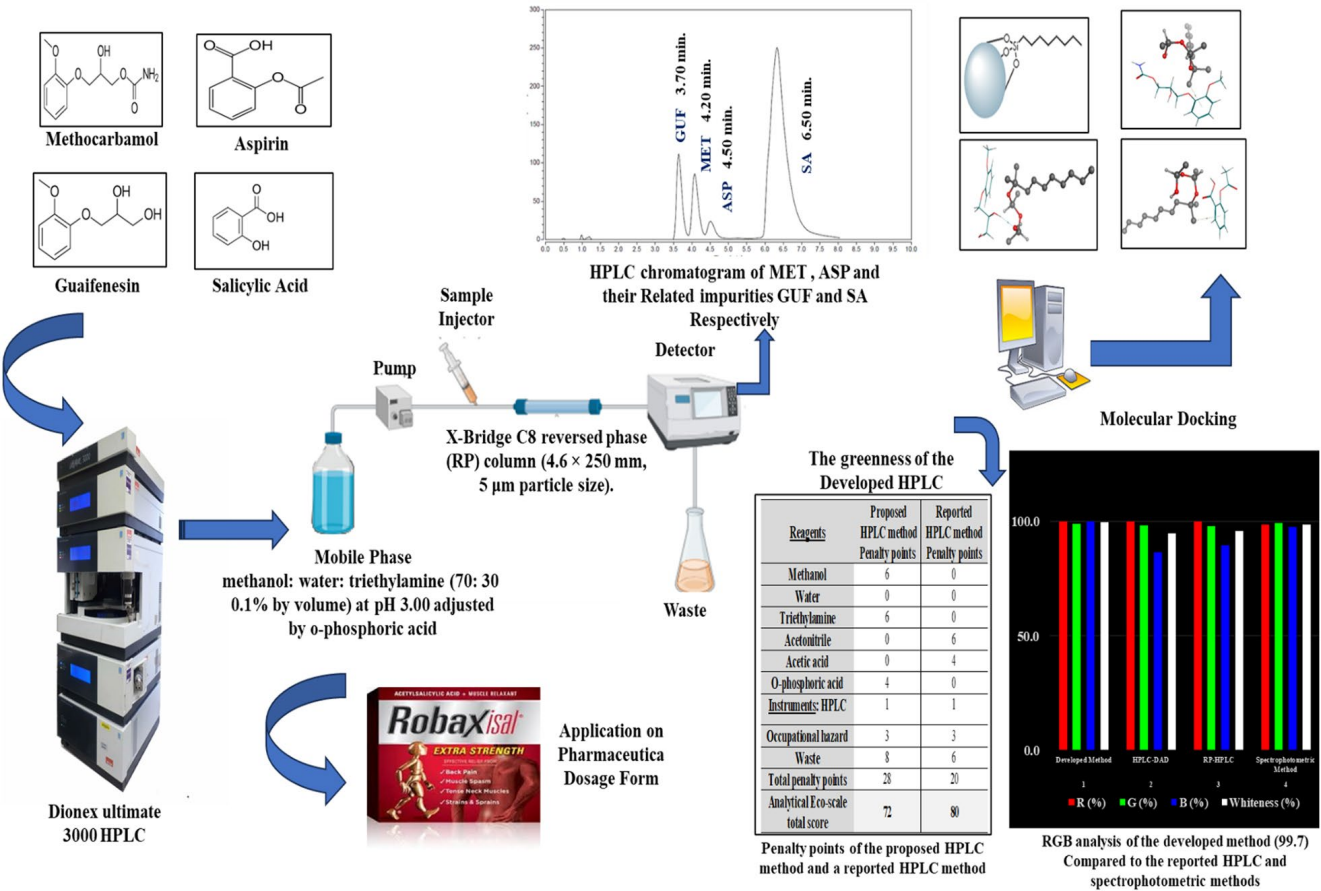

**Supplementary Information:**

The online version contains supplementary material available at 10.1186/s13065-025-01500-7.

## Introduction

Analysis of impurities in the pharmaceutical field is one of the most crucial steps to guarantee their safety, effectiveness and quality [1]. Impurities may result from different sources, including products decomposition and manufacturing procedures [2]. As consequence, the development of an eco-friendly analytical procedures to determine the pharmaceutical dosage form in the presence of its impurities is regarded important [3].

HPLC is an extensive and recommended method used for drug analysis at several stages, covering manufacturing and confirming the quality of pharmaceutical dosage formulations [4]. Researchers become more interested in developing environmentally friendly analytical procedures to reduce environmental ecological impact and improve analyst safety and health conditions. Two different approaches were suggested to assess the established method environmentally, White Analytical Chemistry (WAC) and Green Analytical Chemistry (GAC) [5, 6]. In addition to the green factors, WAC considers two other major factors when estimating the method's quality. The additional two factors are the practical (blue) and analytical (red) aspects [7, 8].

One of the most important computational tools to understand and detect the physical foundation of the structure of compounds is the molecular docking technique through studying the system's dynamic growth, which confirms the practical work output. Predicting the elution sequence and estimating the analytes’ interaction with the stationary phase were the primary objectives of the molecular docking technique, which is used nowadays in chromatographic methodology [9]. The results of the suggested method were then verified by identifying which drug would be more and less retained on the stationary phase [10, 11].

The presence of related impurities may reduce the therapeutic efficacy of their drugs and decrease their stability besides producing undesirable adverse effects. So maximum permitted limits for drug-related impurities have been specified by a number of pharmacopoeias [12]. Additionally, the main clinical application for GUF usually includes expectorant usage. Concerning the analgesic potency of SA, it is five times less than ASP because ASP suppresses the biosynthesis of prostaglandins through acetylation of the cyclo-oxygenase enzyme [13]. The levels of GUA and SA should not be higher than 1% and 3% for MET and ASP respectively, according to the USP [14].

**Fig. 1:**
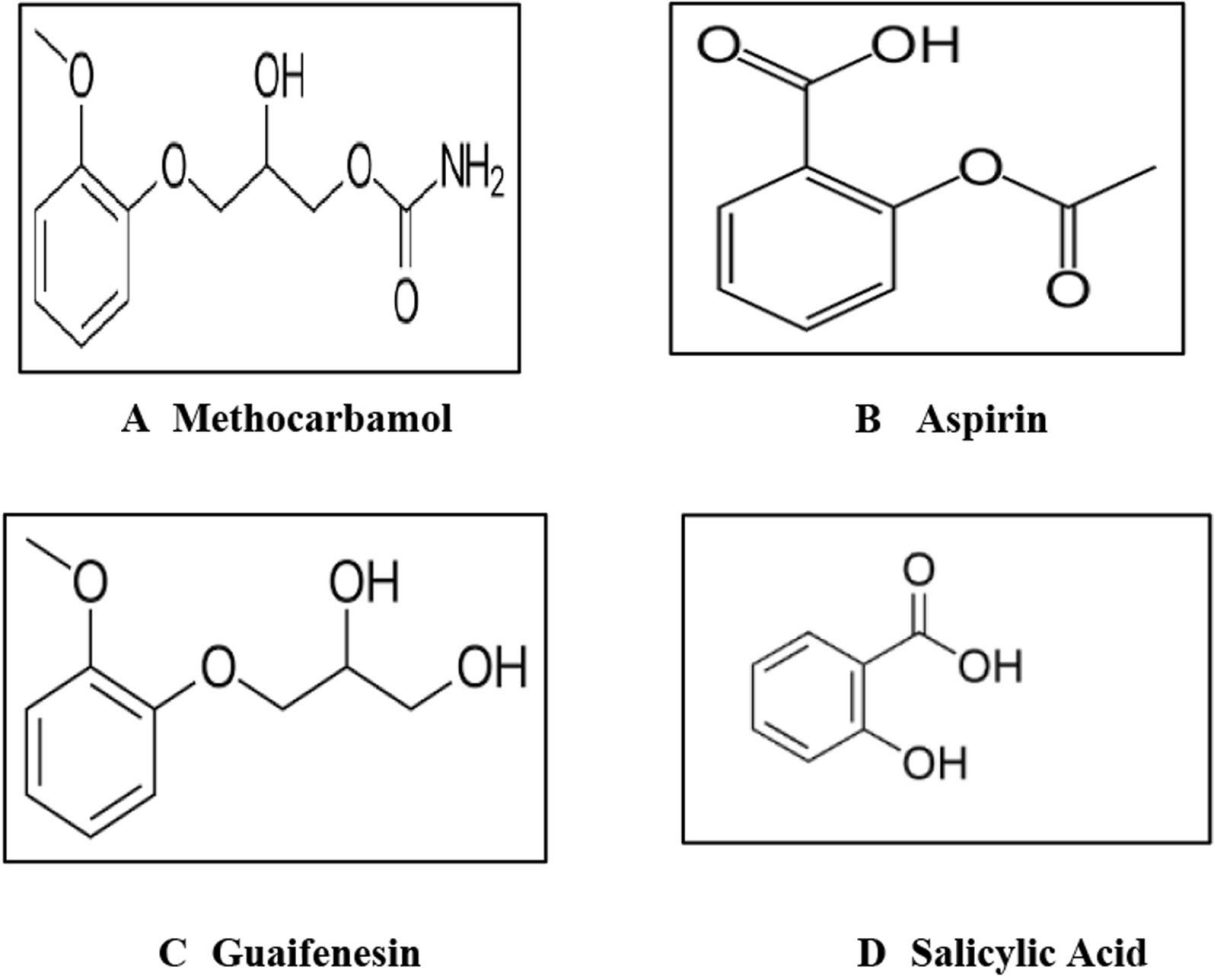
**A** Methocarbamol. **B** Aspirin. **C** Guaifenesin. **D** Salicylic Acid

MET (Fig. 1A) is a 3-2-methoxyphenoxy-1,2-propanediol 1-carbamate. It acts as a muscle relaxant to relief skeletal muscle spasms [15]. Various analytical techniques have been published to quantify methocarbamol individually or in the presence of other drugs, including spectrofluorimetric method [16], electrochemical method [17], spectrophotometric method [18], chromatographic methods [19–22] and TLC-densitometric method [23].

ASP (Fig. 1B) is a 2-Acetyloxy benzoic acid. It acts as analgesic and antipyretic [24, 25]. MET is commonly co-formulated or co-administered with analgesics such as ASP, paracetamol, diclofenac and ibuprofen in order to relieve muscle spasms [26]. GUF and SA (Fig. 1C and D) are reported as impurities through United State pharmacopeia (USP) for MET and ASP, respectively [14]. In addition, GUA and SA are considered to be starting materials in MET and ASP synthesis, respectively, however being as byproducts of their hydrolysis.

The combination of MET and ASP in pharmaceutical dosage form has been utilized as skeletal muscle relaxants and confirmed to be highly effective therapeutic medications to treat muscular spasms, as well as for use as surgical anesthetics. The increasing prevalence of skeletal muscle relaxants in medicine triggered the application of a simple, precise, accurate, sensitive method for analyzing the concentration in pure form and pharmaceutical dosage formulation [26].

Several techniques were reported through USP to determine MET and ASP individually such as spectrophotometric, chromatographic and titrimetric methods [14]. Few analytical techniques have been covered in literatures to determine MET and ASP such as chromatographic [27] and spectrophotometric methods [28]. Currently only one HPLC method has been published for the simultaneous estimation of MET, ASP and their related impurities [29].

Different approaches were utilized to assess the greenness of procedures, including the Analytical Eco-Scale [30] and AGREE [31, 32]. Furthermore, our main objective in the present research was to perform a green, advanced and sensitive HPLC method with low retention times to separate the four intended compounds in one chromatographic run not only in pure powder form but also in spiked human plasma to provide a significant improvement and primary advantage above the only one reported approach [29]. In addition to molecular docking was implemented to investigate the elution performance of the four intended compounds, as well as their interactions with the stationary phase. Furthermore, the greenness and whiteness profiles of developed method were evaluated.

## Experimental

### Apparatus

Dionex Ultimate 3000 HPLC device, provided with a diode array detector and an autosampler for injection. The device is complemented by a tetra solvent delivery pump (Massachusetts, USA). The chromatographic separation was achieved using X-Bridge C_8_ reversed phase (RP) column (4.6 × 250 mm, 5 μm particle size).

### Software

Molecular docking studies were performed using Molecular Operating Environment 2024.02 (Chemical Computing Group, Montreal, QC, Canada).

### Chemicals and solvents

#### Standards and reagents

Standards of MET, ASP, GUF and SA were provided from (Sigma-Aldrich Co, Germany) with purities of about 99.66%, 99.85%, 99.55% and 99.32%, respectively. Methanol of HPLC grade was purchased (Fisher Co., Germany). De-ionized water was utilized. Human plasma was obtained from the blood bank, VACSERA (Holding Company for Biological Products and Vaccines), Egypt.

#### Pharmaceutical formulation

Robaxisal extra strength® coated tablets were acquired from GlaxoSmithKline Consumer HealthCare ULC (GSK) company, Canada. Tablets claimed to contain 400.00 mg MET and 500.00 mg ASP as active ingredients (Batch No. PAA168448).

#### Stock and working solutions

Weights corresponding to 10.00 mg of GUF, MET, ASP, and SA were individually transferred into four separate 10 mL volumetric flasks. The volume in each flask was then adjusted with methanol to achieve final stock solutions of 1.00 mg mL^−1^ for each drug. Subsequently, working solutions for the studied drugs were prepared freshly through appropriate dilution of the previously prepared stock solutions**.**

## Procedures

### Chromatographic conditions

The adequate separating conditions were achieved using X-Bridge C_8_ RP column (4.6 × 250 mm, 5 μm particle size). Optimum separation was achieved utilizing methanol: water: triethylamine (70: 30, 0.1% by volume) at pH 3.00 adjusted by o-phosphoric acid as a mobile phase with flow rate of 2.00 mL min^−1^ and UV detection at 254.00 nm.

### Construction of calibration curves

Concentration ranges of 0.30–50.00 μg mL^−1^, 1.00–300.00 μg mL^−1^, 10.00–500.00 μg mL^−1^ and 0.10–50.00 μg mL^−1^ for GUF, MET, ASP and SA, respectively were established by transferring aliquots from their stock solutions (1.00 mg mL^−1^) into four distinct sets of 50 mL volumetric flasks, which were then completing the volume with methanol. The chromatograms were then recorded and the peak areas for each drug were computed and recorded. Calibration curves were constructed with the corresponding concentrations of each drug and the regression equations were then recorded and computed.

### Application of laboratory prepared mixtures

Several mixtures containing different ratios of MET, ASP and their related impurities were prepared using their respective stock solutions. The prepared solutions were then analyzed using the previously mentioned chromatographic conditions. The concentrations were calculated from the corresponding regression equations for each drug.

### Application to pharmaceutical formulation and standard addition technique

Ten tablets of Robaxisal extra strength® tablet containing MET and ASP were weighed and finely powdered then the average weight of one tablet was calculated. One tablet contains amount equivalent to 400.00 mg and 500.00 mg of MET and ASP, respectively was weighted then transferred into two separate 100 mL volumetric flasks, dissolved in 70 mL of methanol, and sonicated well for 30 min then the volume was completed to the mark. The prepared solutions were then diluted to obtain final concentrations equivalent to 100.00 and 125.00 μg mL^−1^ of MET and ASP, respectively. Then the standard addition approach was applied by adding three known concentrations of the standards of each drug. The added concentrations were selected to be approximately lower, equal and higher than the previously prepared dosage form concentrations.

### Application to spiked human plasma

In two separate 10 mL volumetric flasks, one milliliter of human plasma was added and spiked with a known amount of MET and ASP standards, then the volume was competed using methanol. The area under the curve was recorded and the concentrations of the studied drugs were determined from the corresponding regression equations.

### Molecular docking studies

To rationalize our experimental results, a molecular docking study for methocarbamol (MET) and aspirin (ASP) as well as their related impurities; guaifenesin (GUF) and salicylic acid (SA) with X-Bridge C_8_ reversed phase (RP) column was performed using Molecular Operating Environment 2024.02 (Chemical Computing Group, Montreal, QC, Canada). The structures of C_8_ were built by MOE Builder function. MOE Quick Prep protocol was used to add hydrogen and minimize the structure. The 2 drugs and their impurities were drawn by Chem Bio Draw Ultra Ver.14 Suite (PerkinElmer, Waltham, MA, USA); its potential energy was diminished applying the proper force field AMBER 10 (University of California, San Francisco, CA, USA). The default docking protocol implemented in MOE was applied. Conformations of ligand were fitted in the position with the Triangle Matcher method and ordered with the London ΔG scoring function. The produced poses were ranked as per their docking scores. Finally, the best position in each case according to the docking score was chosen.

### Method validation

Validation of the developed chromatographic method was performed according to the ICH guidelines [33]. The assessed parameters were linearity range, accuracy, precision, limit of detection (LOD), limit of quantification (LOQ) and robustness.

## Results and discussion

In the pharmaceutical analysis field HPLC technique is used extensively, but it generates a significant amount of organic toxic waste. In our research work we always give particular consideration to Green Analytical Chemistry (GAC) [34] not only analysis of the pharmaceutical drugs. We focus especially on the methods and resources available to make sample preparation and analytical procedures more environmentally friendly. The substitution of toxic chemicals, technique miniaturization and automation, which enable significantly lower reagent consumption and waste generation are deemed to be the key tenets. These measures also help to minimize or eliminate the adverse impacts of analytical procedures [5]. Our aim was to develop an eco-friendly, accurate and selective chromatographic method to determine the binary mixture of MET muscle relaxant and ASP in their combined pharmaceutical formulation and spiked human plasma besides in presence of their pharmacopeial related impurities GUF and SA. Our method can also be considered a stability indicating assay method (SIAM) [35] as it enables determining MET and ASP as well as their related impurities and degradation products GUF and SA, respectively.

### Method development and optimization:

**Fig. 2:**
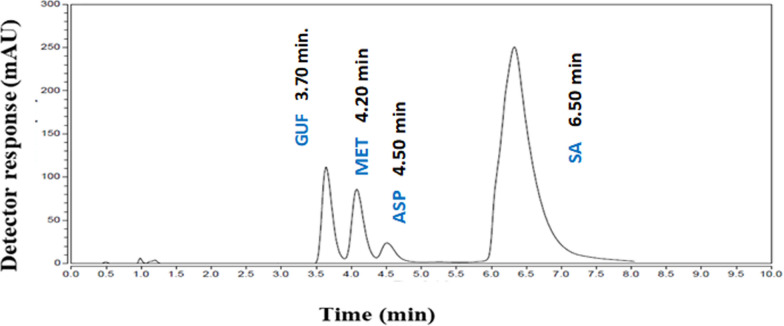
HPLC chromatogram of GUF, MET, ASP and SA

An extensive examination of key factors impacting the effectiveness of the RP-HPLC approach was carried out to assure the method's reliability and performance. A single factor was altered sequentially, while the remaining chromatographic parameters stayed unchanged. Furthermore, method optimization kept the greatest levels of sensitivity and resolution while cutting down on waste production by shortening the time required for chromatographic analysis. Many trials have been conducted on different types of columns to achieve the best possible separation, including X-Bridge C_18_ RP column (4.6 × 250 mm, 5 μm particle size), Equisil C_18_ (5 μm, 4.6 × 250 mm), Kromasil C_18_ (4.6 × 150 mm, 5 μm particle size), Cortecs C_18_ column (4.6 × 50 mm, 2.7 μm particle size) and Cortecs C_8_ column (4.6 × 50 mm, 2.7 μm particle size). Best results were achieved using X-Bridge C_8_ RP (4.6 × 250 mm, 5 μm particle size) column. Concerning the mobile phase, we took care to use safe and eco-friendly solvents as possible as we could, to minimize environmental risks during the technique development stage. Initially, mobile phases composed of methanol: water were tested with different ratios as (50: 50, v/v), (70: 30, v/v), (30: 70, v/v), (60: 40, v/v) at different pH ranges 3.00, 3.50 and 4.00 using o-phosphoric acid. Additionally, mobile phases composed of methanol: 0.1% formic acid (50: 50, v/v), (60: 40, v/v) and (80: 20, v/v) were tried. Moreover, we tried methanol: phosphate buffer at various pH ranges 3.00, 3.50 and 4.50 using o-phosphoric acid in the ratios (50: 50, v/v), (70: 30, v/v) and (30: 70, v/v). Regretfully, the four compounds could not be effectively separated by any of the previous mobile phases as overlapped peaks were obtained. We started to observe improved in the separation by using methanol: water: triethylamine (50: 50, 0.1% by volume), (60: 40, 0.1% by volume) and (70: 30, 0.1% by volume). Mobile phase consisted of methanol: water: triethylamine (70: 30, 0.1% by volume) adjusting pH to 3.00 by o-phosphoric acid produced the ideal resolved peaks. Particularly in terms of lowering the peak tailing, the findings have improved with the addition of trimethylamine [36]. Additionally, many flow rates and UV detection wavelengths have been examined as 1.00, 1.50, 2.00- and 2.50 mL min^−1^ and 210.00, 254.00 and 270.00 nm, respectively. Eventually, best conditions for optimum separation and acceptable system suitability parameters were achieved using X-Bridge C_8_ RP (4.6 × 250 mm, 5 μm particle size) column, methanol: water: triethylamine (70: 30, 0.1% by volume) at pH 3.00 by o-phosphoric acid with 2.00 mL min^−1^ flow rate and UV detection at 254.00 nm, as presented in Fig. 2.

Several system suitability parameters were assessed to the separated peaks, including tailing factor, resolution, retention factor and separation factor. These measurements were then compared to the USP pharmacopeia acceptable reference values [14]. Table 1 illustrates the comparison results, which support the developed method's compliance with the necessary system suitability criteria. The chromatographic method was optimized, leading to an improvement in the separation efficiency and the produced system suitability parameters to analyze GUF, MET, ASP and SA.

**Table 1:** System suitability parameters for the proposed HPLC method

Parameters	GUF	MET	ASP	SA	Acceptable values [14]
Retention time (t_R_) (min)	3.70	4.20	4.50	6.5	–
Resolution (R_S_)	1.25		2.22		> 1
Separation Factor (α)	1.20		1.65		> 1
Symmetry Factor (T)	1	1	1	1.08	= 1 for typical symmetric peak
Retention Factor (Kˋ)	1.96	2.36	2.60	4.20	The higher the retention factor, the longer the retention time
Column Efficiency (N)	5476	7056	5184	1600	Increase efficiency of separation
HETP^*^	4.57 × 10^–3^	3.54 × 10^–3^	4.82 × 10^–3^	1.56 × 10^–2^	The smaller the value the higher the column efficiency

^*^Height equivalent to theoretical plate (cm/plate)

### Molecular docking studies

**Fig. 3:**
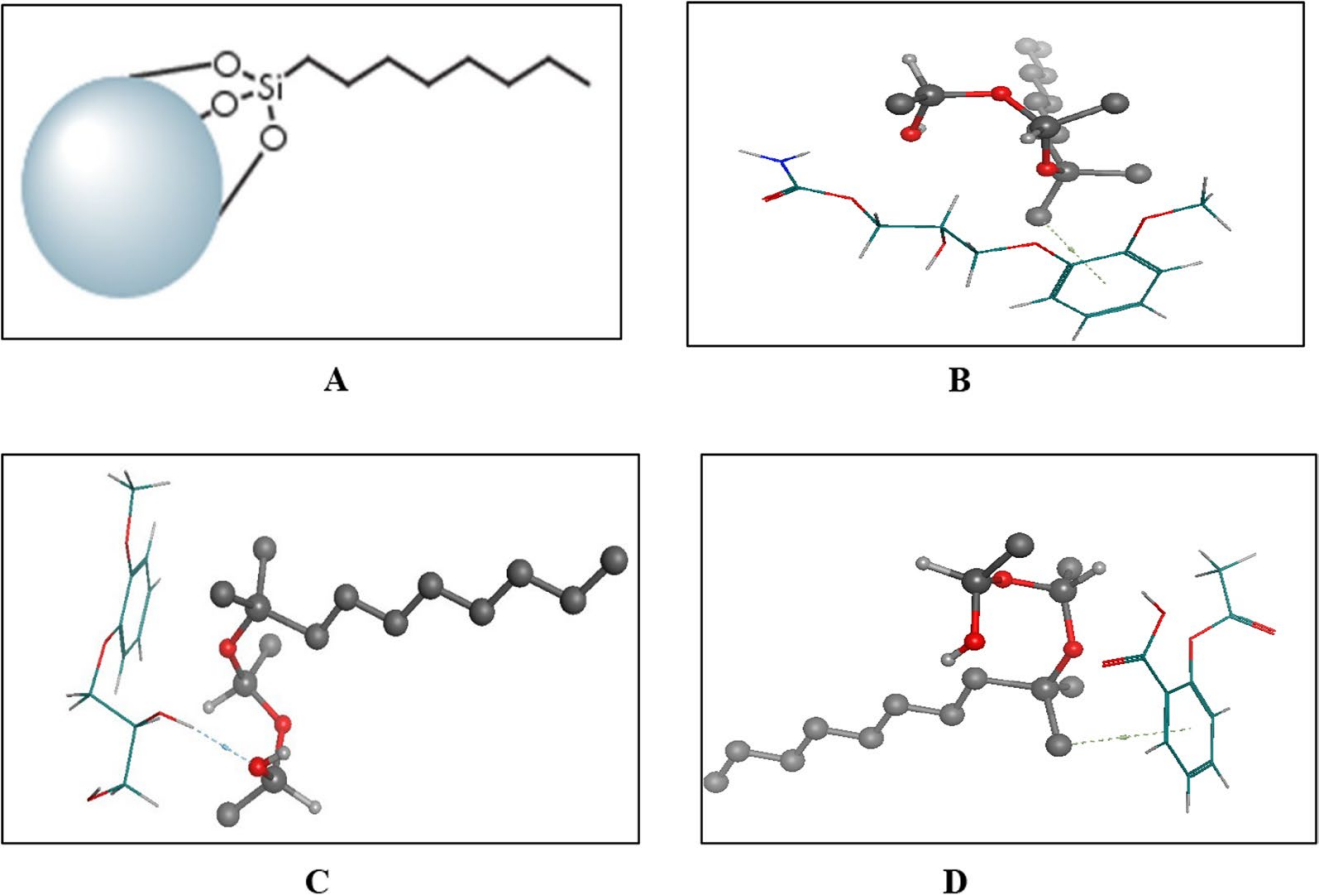
Diagram presents: **A** Composition of X-Bridge C_8_ reversed phase column, **B** 3D interaction of MET and the reversed phase stationary phase Si–O showing hydrophobic interaction, **C** 3D interaction of GUF and the reversed phase stationary phase Si–O showing hydrogen bond interaction, **D** 3D interaction of ASP and the reversed phase stationary phase Si–O showing van der-waals interaction

In this work, the interactions between the drugs and the stationary phase were performed and the binding energies were determined using a reversed phase X-Bridge C_8_ column Fig. 3A, which only had an octa chain that could provide hydrophobic interactions. After simulating the same conditions for MET and its impurity component GUF, we thoroughly examined the resulting trajectories and discovered that both the drug and its impurity component interact with the stationary phase in multiple ways. Polarity affects how pharmaceuticals interact with the column; the least polar medication will be the one that is most retained with the stationary phase, while the most polar drug will be the one that is least retained with the stationary phase. It was discovered that MET forms a hydrophobic interaction, Fig. 3B, However, as shown in Fig. 3C, its impurity elutes first with a binding energy of − 3.00 kcal/mol because it was found to be more polar than the drug in its intact form. Aspirin elutes with a binding energy of − 3.13 kcal/mol because it exhibits less polarity, as seen in Fig. 3D. It also served to validate the results of the suggested method.

### Method validation

In compliance with ICH recommendations [33], the validation scheme was applied to the suggested HPLC method, which include linearity range, accuracy, precision, LOD and LOQ as well as robustness.

#### Range and linearity

A linear relationship was established between the peak area and the corresponding concentration of each drug under the specific experimental conditions in the range of 0.30–50.00 μg mL^−1^, 1.00–300.00 μg mL^−1^, 10.00–500.00 μg mL^−1^ and 0.10–50.00 μg mL^−1^ for GUF, MET, ASP and SA, respectively. The results of the validation parameters of the proposed HPLC method were summarized in Table 2**.**

**Table 2:** Validation parameters of the proposed HPLC method to determine, MET, ASP GUF and SA in pure powder forms

Parameters	MET	ASP	GUF	SA
Linearity				
Slope	0.11	0.12	0.10	1.03
Intercept	0.20	0.68	0.09	1.16
Correlation coefficient (r)	0.9999	0.9999	0.9997	0.9999
Accuracy (Mean ± SD)	101.07 ± 0.89	100.70 ± 0.78	99.69 ± 1.56	100.12 ± 0.70
Precision (%RSD)				
Repeatability^a^	± 0.88	± 0.78	± 1.57	± 0.70
Intermediate precision	± 1.22	± 0.73	± 1.48	± 1.03
Specificity (Mean ± SD)	101.98 ± 1.25	100.89 ± 0.88	97.99 ± 0.90	99.99 ± 1.10
Specificity and selectivity				Resolution values (1.25 & 2.22)
Range (µg mL^−1^)	1.00–300.00	10.00–500.00	0.30–50.00	0.10–50.00
Robustness (%RSD)				
Resolution (R_S_)	1.38	1.18	1.07	0.94
Symmetry factor (T)	1.01	1.01	1.00	1.94
LOD^c^ (µg mL^−1^)	0.31	5.11	0.08	0.03
LOQ^c^ (µg mL^−1^)	0.94	9.27	0.25	0.09

^a^Intraday precision (the RSD of three different concentrations (1.00, 10.00, 50.00 μg mL^−1^ for GUF, 10.00, 100.00, 300.00 μg mL^−1^ for MET, 10.00, 200.00, 500.00 μg mL^−1^ for ASP and 1.00, 10.00, 50.00 μg mL^−1^ for SA) / 3 replicates each, within the same day)

^b^Interday precision (the RSD of three different concentrations (1.00, 10.00, 50.00 μg mL^−1^ for GUF, 10.00, 100.00, 300.00 μg mL^−1^ for MET, 10.00, 200.00, 500.00 μg mL^−1^ for ASP and 1.00, 10.00, 50.00 μg mL^−1^ for SA)/3 replicates each, repeated on 2 successive days)

^c^Limit of detection and quantitation are determined via calculations, LOD = (SD of the response/slope) × 3.3; LOQ = (SD of the response/slope) × 10

#### Accuracy

Accuracy was assessed by calculating the percentage recovery for three replicates of three different concentrations covering the linearity range for the studied drugs as presented in Table 2**.**

#### Precision

Precision was evaluated by the analysis of three concentration levels within the day and for three successive days for both repeatability and intermediate precision, respectively. The results were presented in Table 2.

#### LOD and LOQ

Regarding the limits of quantification (LOQ) and limit of detection (LOD), they were calculated based on the standard deviations of intercepts. Table 2 shows results of limit of detection and quantitation that are determined via calculations, LOD = (SD of the response/slope) × 3.3; LOQ = (SD of the response/slope) × 10.

#### Robustness

In order to assess the robustness of the suggested HPLC method, we examined the impact of minute deliberate adjustments to the resolution (Rs) and symmetry factor (T) of the experimental condition. Among these variations were different methanol composition of 70.00 ± 5.00% as the mobile phase, pH values change (3.00 ± 0.20), wave length change (254.00 ± 5 nm) and temperature change (20.00 ± 5.00 ℃). The values of each parameter were altered while the others remained unchanged. The fact that these small modifications to the experimental setup had no discernible effect on the reproducibility or repeatability of the procedure as illustrated in Table 2 according to the obtained relative standard deviations (%RSD) which indicates how reliable the developed method is.

#### Specificity

Several laboratory prepared mixtures containing different concentrations of the studied drugs and their related impurities were evaluated in order to ascertain the method specificity. Satisfactory results were obtained indicating the high selectivity of the proposed method as presented in Table 2.

### Application to pharmaceutical formulation

The recommended HPLC method was effectively implemented to ascertain MET and ASP in their pharmaceutical formulation Robaxisal extra strength® tablet. Table 3 displays the acquired results that demonstrate a strong correlation with the labeled values of the two medications in their tablet dosage form, showing the precision and selectivity of the presented method. No interference from excipients was revealed when the standard addition technique was applied to assess the validity of the method for the dosage form.

**Table 3:** Determination of MET and ASP in Robaxisal extra strength® tablet by the proposed HPLC method and application of standard addition technique

Pharmaceutical Formulation	Drug	Proposed method % Recovery (mean ± SD)	Standard Addition			
			Taken (μg mL^−1^)	Added (μg mL^−1^)	Standard found (μg mL^−1^)	% Recovery of added
Robaxisal extra strength® tablet labeled to contain 400.00 mg MET and 500.00 mg ASP/ tablet (Batch No. PAA168448)	MET	100.42 ± 1.66 ^a^	100.00	50.00	49.31	98.61
			100.00	100.00	100.13	100.13
			100.00	150.00	152.85	101.90
			Mean R% ± SD		100.21 ± 1.65	
	ASP	99.53 ± 1.21 ^b^	125.00	50.00	49.76	99.51
			125.00	100.00	100.02	100.02
			125.00	200.00	202.01	101.00
			Mean R% ± SD		100.18 ± 0.76	

^a^Results of analysis 100.00 μg mL^−1^ of MET three times

^b^Results of analysis 125.00 μg mL^−1^ of ASP three times

### Application to spiked human plasma

The proposed method offered a rapid, selective and sensitive approach for monitoring MET and ASP levels in spiked human plasma that is suitable for pharmacokinetic or bioequivalence research studies. We applied direct injection method without time consuming extraction procedures [37–41], showing promising results in Table 4.

**Table 4:** Determination of MET and ASP in spiked plasma by the developed HPLC method

Drug		MET	ASP
		Recovery % ± SD	Recovery % ± SD
Spiked Human Plasma	101.07 ± 0.89 ^a^		100.70 ± 0.78 ^b^

^a^Results of analysis 100.00 μg mL^−1^ MET three times

^b^Results of analysis 100.00 μg mL^−1^ of ASP three times

In order to compare the data resulted from both the proposed HPLC method and the reported one [29], the statistical F-test and Student t-test were applied. Table 5 illustrates that the measured F- and t-values were found to be below the critical values, indicating no significant difference with respect to accuracy.

**Table 5:** Statistical comparison of the results obtained by the proposed HPLC method and a reported HPLC method [29] to determine MET, ASP, GUF and SA in pure powder form

Drug	MET		ASP		GUF		SA	
Parameters	Reported method*	Proposed method	Reported method*	Proposed method	Reported method*	Proposed method	Reported method*	Proposed method
Mean	100.12	101.07	100.98	100.70	100.11	99.69	101.02	100.12
SD	1.77	0.89	1.52	0.78	1.62	1.56	0.87	0.70
RSD%	1.77	0.88	1.51	0.78	1.62	1.57	0.86	0.70
n	6	6	6	6	6	6	6	6
Variance	3.13	0.79	2.31	0.61	2.62	2.43	0.76	0.49
F-value (p = 0.05)	–	3.96 (4.28)	–	3.79 (4.28)	–	1.08 (4.28)	–	1.55 (4.28)
Student’s t-test	–	1.18 (2.44)	–	0.40 (2.44)	–	0.46 (2.44)	–	1.97 (2.44)

The figures between parenthesis are the corresponding theoretical values of F & t at p = 0.05

^*^HPLC method C_18_ column using isocratic elution system of diluted acetic acid (pH 3.2): acetonitrile at the ratio of 79: 21, v/v, at a flow rate of 1 mL min^−1^. Detection was achieved at 233 nm for MET, GUF and SA and at 273 nm for ASP

### Greenness assessment of the developed method

**Fig. 4:**
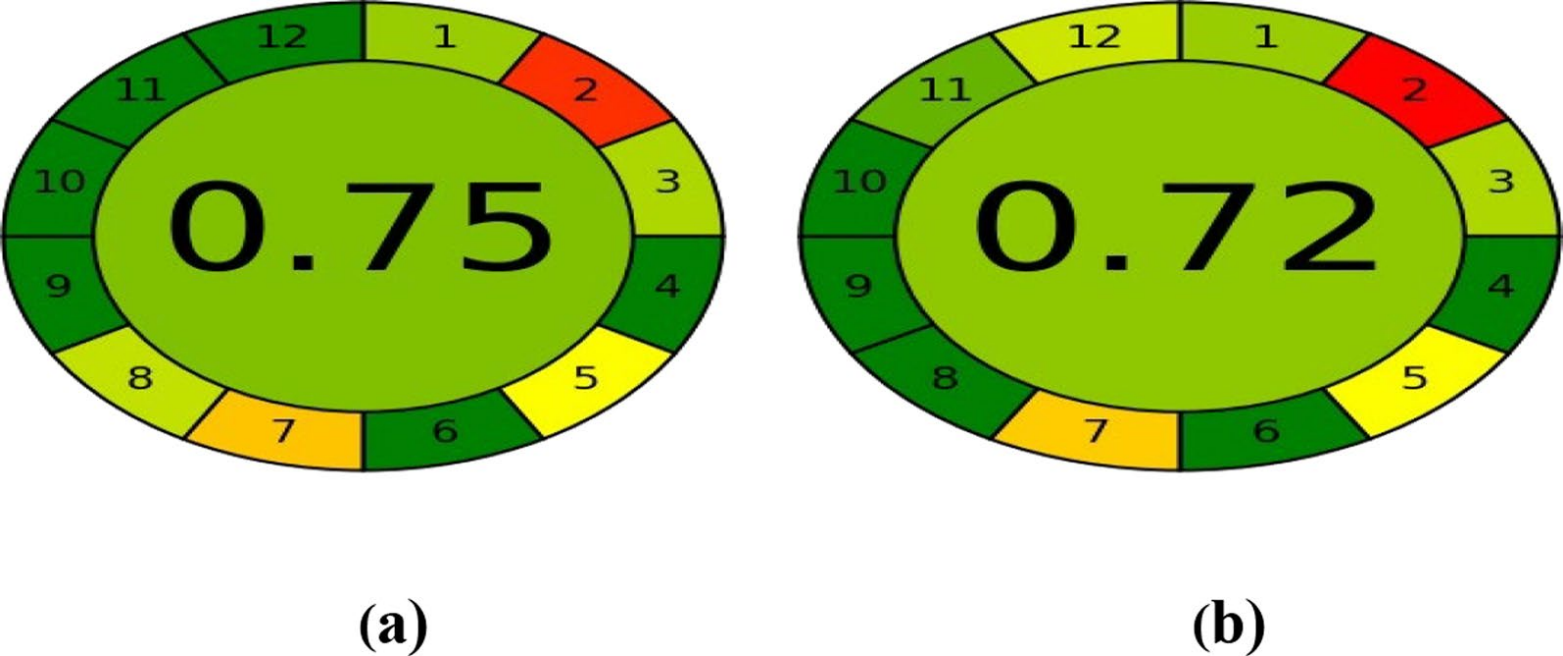
AGREE green profile assessment of **a** the developed HPLC method to determine MET and ASP, **b** the reported HPLC method to determine MET and ASP

Nowadays, researchers focus on establishing green analytical chemistry (GAC) rather than developing validated analytical method. The developed method was evaluated utilizing two green analytical assessment tools namely; Analytical Eco-Scale and AGREE approaches [30, 31]. The first applied approach is the Analytical Eco-Scale, it is a thorough tool for evaluating analytical procedures in a semi-quantitative manner. Table 6 illustrates the detailed total penalty points for our proposed HPLC method versus a reported HPLC one [29]. In order to evaluate the analytical procedure, a value of penalty points is assigned to each step and subtracted from a base of 100. The second approach is AGREE which is considered one of the recent greenness tools of assessment. As presented in Fig. 4, the circle becomes green when the score comes close to one. The suggested method received a score of 0.75, indicating greater greenness compared to reported HPLC and spectrophotometric methods [29]. Finally, based on the obtained data, the proposed approach yielded a green analytical profile.

**Table 6:** Penalty points for using the proposed HPLC method and a reported HPLC method [29]

Reagents	Proposed HPLC method Penalty points	Reported HPLC method Penalty points
Methanol	6	0
Water	0	0
Triethylamine	6	0
Acetonitrile	0	6
Acetic acid	0	4
O-phosphoric acid	4	0
*Instruments*: HPLC	1	1
Occupational hazard	3	3
Waste	8	6
Total penalty points	28	20
Analytical Eco-scale total score	72	80

### Whiteness assessment of the developed method

**Fig. 5:**
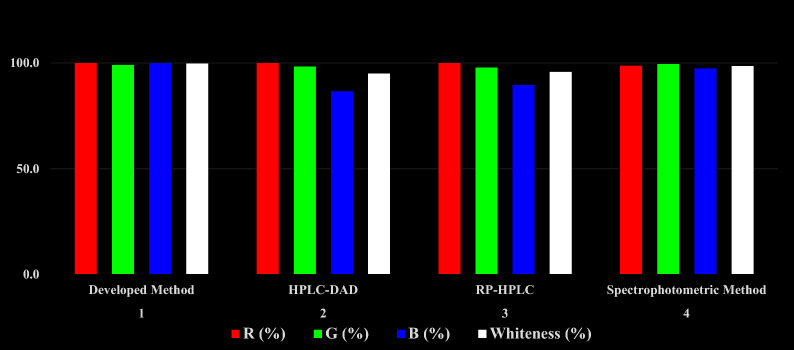
Comparison of the results obtained from RGB analysis for the developed HPLC method with the reported HPLC and spectrophotometric methods

The green aspects of the proposed HPLC method were assessed using the whiteness assessment approach [7, 8]. Red Green Blue (RGB) is considered as one of the most published multi-criteria assessments techniques and the results were presented in Table 7 and Fig. 5. Through the computational and obtained results for whiteness assessment, our developed HPLC method is considered greener and more eco-friendly than the reported HPLC and spectrophotometric methods [27–29].

**Table 7:**
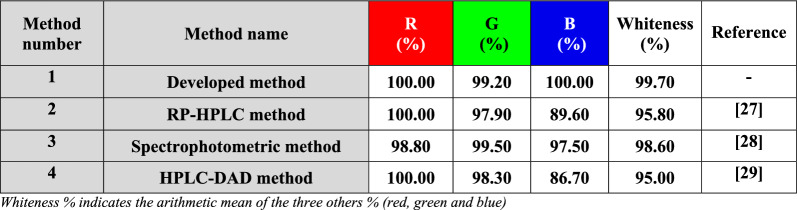
RGB profiles of the developed HPLC method and other reported methods

Whiteness % indicates the arithmetic mean of the three others % (red, green and blue)

Table 8 provided a summary of some of the key outcomes from the suggested approach and the former mentioned HPLC method [29]. Eventually, our presented method is more sensitive, economic and time-saving with a shorter run time. Besides, it can detect a wider range and lower concentrations of the intended drugs with wider application in spiked human plasma compared to the reported HPLC method. The developed work also has low impact on the ecosystem. Although the results obtained from the Analytical Eco-Scale were better than our suggested method, AGREE and Whiteness assessments showed superior outcomes for the proposed method. Additionally, molecular docking was novelly employed to ascertain the elution arrangement for the four studied compounds. Consequently, the worked HPLC method describes an eco-friendly, sensitive and cost-effective stability indicating assay to simultaneously determine MET and ASP as well as their related substances GUF and SA, respectively.

**Table 8:** Comparison of the results obtained by the proposed HPLC and the reported one [29]

Parameters	Proposed HPLC method				Reported HPLC method			
	MET	ASP	GUF	SA	MET	ASP	GUF	SA
Range (µg mL^−1^)	1.00–300.00	10.00–500.00	0.30–50.00	0.10–50.00	2.00–150.00	25.00–450.00	0.40–3.00	0.20–27.00
R_t_ (min)	4.20	4.50	3.70	6.50	5.07	5.81	4.15	7.84
Total run time (min)	8				9			
Analytical Eco-scale (penalty points)	72				80			
Whiteness evaluation (%)	99.70				95.00			
AGREE	0.75				0.72			

## Conclusion

Through several analytical trials, the proposed research offered high sensitivity, precision and accuracy, which makes it suitable for the routine analysis besides the simultaneous separation and quantification of MET, ASP and their related impurities and degradation products GUF and SA, respectively in their pure form and in their combined pharmaceutical formulation. Furthermore, the developed approach has been successfully applied to determine the studied drugs in spiked human plasma. Utilizing HPLC technique to separate MET, ASP and their related impurities GUF and SA provided several advantages over the published method in reducing analysis time and thus reduce solvents consumption through separation of the studied drugs all in a single run. Additionally, by utilizing molecular dynamic simulation, the separation power was linked to the computational outcomes. Moreover, in addition to offering these benefits, the suggested HPLC approach is environmentally friendly and green. Analytical Eco-Scale and AGREE approaches beside whiteness assessment were applied to measure the environmental impact for the present work. Consequently, the discussed HPLC method offers more rapid analysis and more economic additionally environmentally sustainable.

## Supplementary Information

Supplementary Material 1.

## Data Availability

All data generated or analyzed during this study are included in this article.
