# Supplementary material for: Green chromatographic approach to determine methocarbamol, aspirin and their related impurities in their combined pharmaceutical formulation and human plasma: with computational evaluation
Source: BMC Chem. 2025 May 21;19(1):136. doi: 10.1186/s13065-025-01500-7 (PMC12093686; doi:10.1186/s13065-025-01500-7)
Supplement: Supplementary file 1 — Supplementary Material 1. [file 13065_2025_1500_MOESM1_ESM.docx]

**
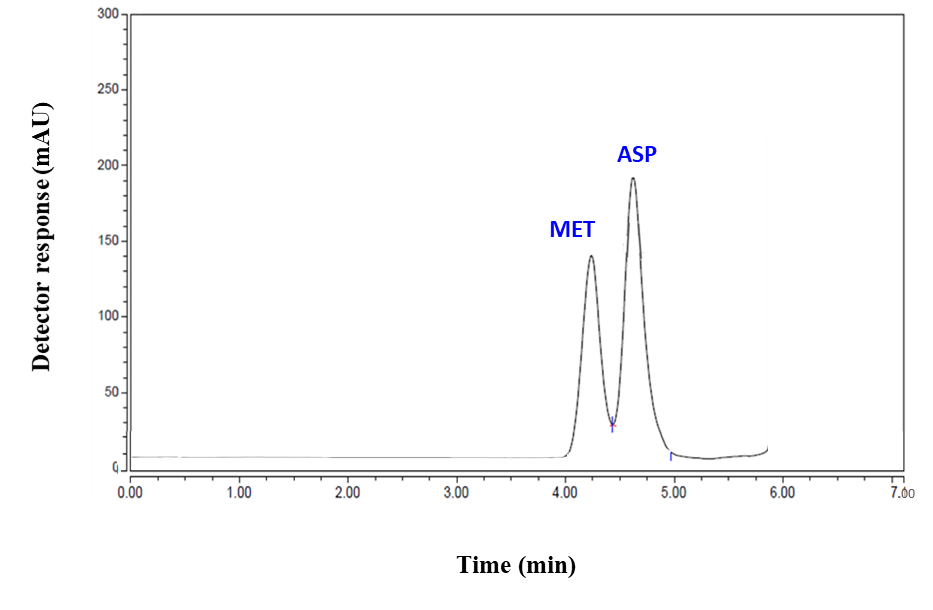
**

**Supplementary S1: HPLC chromatogram of 100 µg mL^-1^ MET and 125 µg mL^-1^ ASP in their pharmaceutical formulation (Robaxisal extra strength^®^ tablet)**


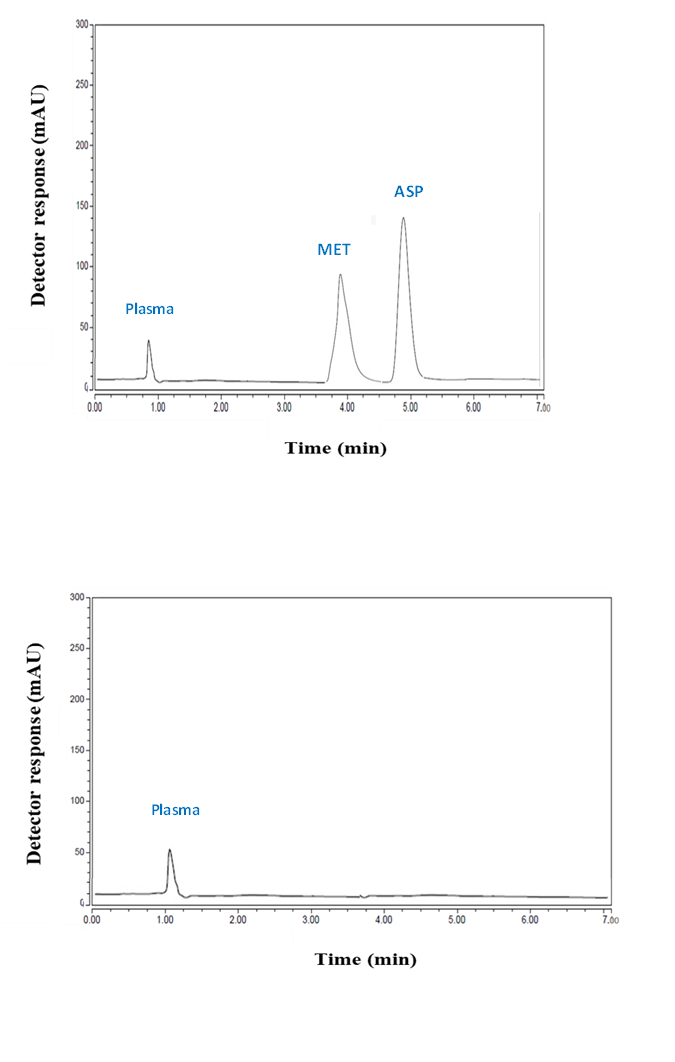


**Supplementary S2: HPLC chromatogram of drug free plasma.**


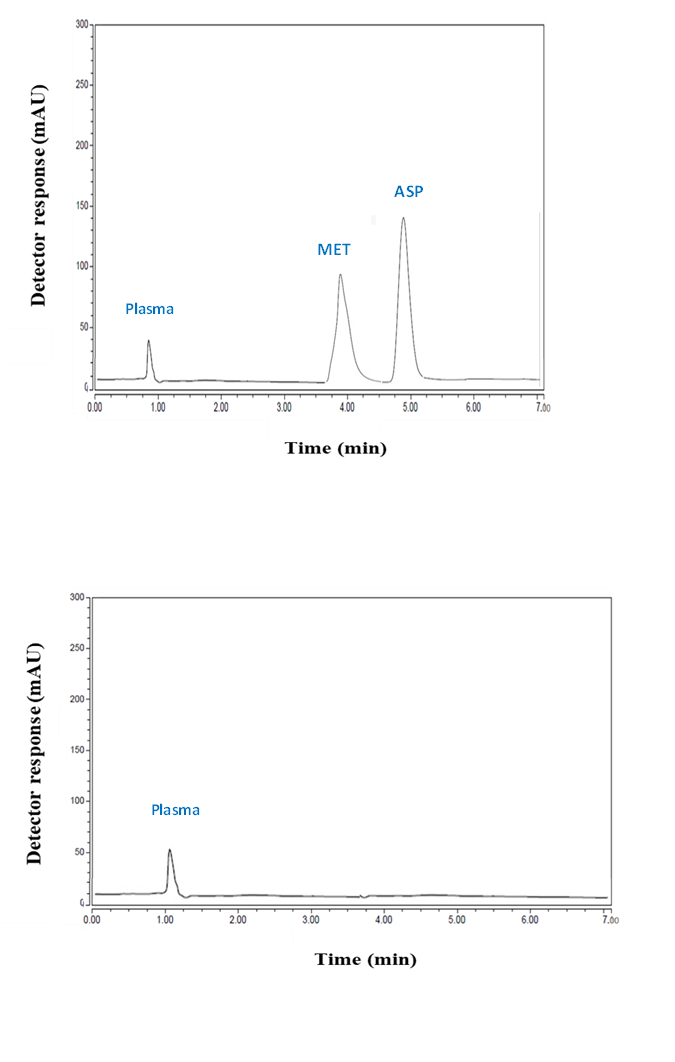


**Supplementary S3: HPLC chromatogram of plasma spiked with MET and ASP.**
